# TOTAL AND POST-DISCHARGE 30-DAY EPISODE PAYMENTS FOR BENEFICIARIES WITH ALZHEIMER’S AND DEMENTIA

**DOI:** 10.1093/geroni/igz038.1699

**Published:** 2019-11-08

**Authors:** Neil Kamdar, Elham Mahmoudi

**Affiliations:** 1 University of Michigan, Ann Arbor, Michigan, United States; 2 University of Michigan, Department of Family Medicine, Ann Arbor, Michigan, United States

## Abstract

There exists lack of evidence regarding incremental post-discharge cost and utilization of healthcare services for older adults with Alzheimer’s and dementia (AD). We quantified episode payments associated with AD vs. non-AD 30 days after medical or surgical procedures. We utilized administrative claims between January 2012 and June 2017 from the Michigan Value Collaborative (MVC) across 31 different medical and surgical services. We identified all patients with any AD diagnosis code throughout their enrollment using ICD-9-CM, ICD-10-CM codes. We price standardized 30-day episode payments and split them based on patient setting. Payments were risk adjusted and winsorized at the 99th /1st percentile. Propensity score matching using calipers without replacement adjusted for clinically relevant surgical and medical procedures, HCCs, insurance type, and age to control for selection bias. We identified 66,676 AD episodes and 656,235 non-AD episodes. After propensity score matching, there were 58,485 AD and non-AD episodes with significant differences in total episode payments of ($22,378 vs. $19,595, 95% CI Diff: ($2,658, $2,910)). Post-acute care and readmission payments were significant ($4,561 vs. $3,272, 95% CI Diff: ($1,235, $1,342)) and ($1,807 vs. $1,165, 95% CI Diff: ($595, $691)), respectively. AD episodes had a higher readmission rate (21.6% vs. 14.8%, p<0.0001). County variation in payments for AD episodes was substantial (Median: $4,370, Range: $3,881). AD patients are at higher risk of readmission and more resource intensive to hospitals and health systems. Examining drivers of post-discharge cost variation can influence practice pattern changes in management of AD patients.

